# An approach to identify people with mental illness that can be expected to benefit from integrated community care in Germany

**DOI:** 10.1192/j.eurpsy.2022.1572

**Published:** 2022-09-01

**Authors:** F. Meixner, R. Kilian, A. Müller-Stierlin

**Affiliations:** 1 Ulm University, Department Of Psychiatry Ii, Bezirkskrankenhaus Günzburg, Günzburg, Germany; 2 Ulm University, Institute Of Epidemiology And Medical Biometry, Ulm, Germany

**Keywords:** severe mental illness, Community-based mental health care, Integrated community care, Assessment

## Abstract

**Introduction:**

Although integrated community care programs specifically tailored to patients with severe mental illness (SMI) are available, recent studies show that these programs are not always provided to the population which would benefit the most from it.

**Objectives:**

Aims of this study were the selection of clinical and psychosocial characteristics and the development of a screening algorithm indicating the need for integrated community care services in people with mental disorders.

**Methods:**

Data of an observational longitudinal study including N=511 participants has been used to examine the hypothesized determinants. At baseline, self-reported empowerment has been assessed via the EPAS and psychosocial impairment and perceived needs have been rated by research workers via the HoNOS and the CAN, respectively. Use of integrated community care services was defined as at least four appointments with service providers over six months and has been recorded via the CSSRI twelve to 18 months after baseline. Mixed-effects regression analyses have been performed to test the predictive value of the hypothesized determinants and marginal predictions were used to define cut-offs for the assessment tool.

**Results:**

EPAS, HoNOS and CAN scores each proved to be significant predictors for using integrated community care services. Cut-off scores for each predictor are presented, forming practical assessment guidelines for future studies.

**Conclusions:**

A screening tool and an algorithm for the identification of mentally ill patients who can be expected to benefit from integrated community mental health care programs is available for the German health care system.

**Disclosure:**

No significant relationships.

